# Protective effect of bone marrow mesenchymal stem cell-derived exosomes on cardiomyoblast hypoxia-reperfusion injury through the HAND2-AS1/miR-17-5p/Mfn2 axis

**DOI:** 10.1186/s12872-023-03148-4

**Published:** 2023-03-07

**Authors:** Qiang Li, Yanling Bu, Haifeng Shao, Wenhua Li, Di Zhao, Jian Wang

**Affiliations:** 1 Department of Cardiology, The Third Affiliated Hospital of Qiqihar Medical University, 27 Taishun street, Tiefeng District, Qiqihar, 161099 China; 2 Department of Ultrasonography, The Third Affiliated Hospital of Qiqihar Medical University, Qiqihar, 161099 China

**Keywords:** Exosome, HAND2-AS1/miR-17-5p/Mfn2, H/R damage, Oxidative stress, Inflammation

## Abstract

**Background:**

The exosomes (exos) of bone marrow mesenchymal stem cells (BMSCs) play an important therapeutic role in repairing myocardial injury. The purpose of this study was to explore how the exos of BMSCs can alleviate the myocardial cell injury caused by hypoxia/reoxygenation (H/R) through HAND2-AS1/miR-17-5p/Mfn 2 pathway.

**Methods:**

Cardiomyocytes H9c2 were damaged by H/R to mimic myocardial damage. Exos were gained from BMSC. The content of HAND2-AS1 and miR-17-5p was assessed by RT-qPCR. Cell survival rate and apoptosis were estimated by MTT assay and flow cytometry. Western blotting was used to detect the expression of protein. The contents of LDH, SOD, and MDA in the cell culture were detected by commercial kits. The luciferase reporter gene method confirmed the targeted relationships.

**Results:**

In H9c2 cells induced by H/R, the level of HAND2-AS1 declined and the expression of miR-17-5p was elevated, but their expression was reversed after exo treatment. Exos improved the cell viability, declined cell apoptosis, controlled the oxidative stress, and repressed inflammation, thus attenuating the damage of H9c2 induced by H/R, whereas, the knockdown of HAND2-AS1 partly alleviated the impacts of exos. MiR-17-5p played the opposite role to HAND2-AS1 on H/R-injured myocardial cells.

**Conclusion:**

Exos derived from BMSC could alleviate H/R-induced myocardial injury by activating HAND2-AS1/miR-17-5p/Mfn2 pathway.

**Supplementary Information:**

The online version contains supplementary material available at 10.1186/s12872-023-03148-4.

## Introduction

Acute myocardial infarction (AMI) and heart failure have become worldwide health burdens [1]. Timely reperfusion therapy can significantly improve the outcome of patients with AMI, but there are still quite a few patients who cannot get effective treatment timely. Moreover, some patients with myocardial infarction cannot avoid heart failure even if they get timely reperfusion treatment [2]. The latter is thought to be related to the mechanisms of ischemia-reperfusion injury and apoptosis [3]. Therefore, the mechanism of ischemia-reperfusion injury is crucial for the prognosis of patients.

Repairing and regenerating damaged cardiomyocytes by stem cells is the key research content of various heart diseases, but the efficiency of differentiation of mesenchymal stem cells (MSCs) into damaged cells is very low and the survival time is short. The differentiation and effectiveness in ischemic and hypoxia environment are still controversial [4]. However, the therapeutic effect of stem cells on myocardial damage is obvious, so the paracrine effect of stem cells has attracted extensive attention. Exosomes (exos) are considered to be an important medium of paracrine [5]. Exo is a kind of vesicle containing a phospholipid bilayer, which is composed of intracellular multi-vesicular body. It fuses with the cell membrane and then releases the vesicles outside the cell [6]. MSCs can secrete exos. Exos-mediated intercellular information exchange is widely involved in many pathophysiological processes of heart diseases [7]. In animal models, the regenerative effect of exos may be comparable to that of parental cells in promoting regeneration and functional recovery [8]. Therefore, it is very important to understand the protective effect of stem cell-derived exos in the process of myocardial injury repair.

Exos, also known as microbubbles, are rich in cytokine proteins, phospholipids, and various RNA [9]. LncRNA plays an intermediate role in the process of exo’s function realization. Exo can deliver specific lncRNA to target cells in a short distance or long distances to play the role of exo [10, 11]. The lncRNA contained in exos is considered as an important regulator of myocardial cells and vascular smooth muscle cells [11]. LncRNA acts on related target molecules to inhibit myocardial ischemia-reperfusion injury. LncRNA HCP5 derived from MSCs is involved in cell development and apoptosis of ischemia/reperfusion (I/R) through the IGF1/PI3K/AKT pathway [12]. In the hypoxia/reoxygenation (H/R) model in vitro, the secretion of lncRNA KLF3-AS1 from MSCs can improve the viability of H9c2 cells by binding STAT5B, thus recovering the myocardial injury [13]. Umbilical MSC-exo-derived lncRNA UCA1 may mitigate H/R lesions by the miR-143/Bcl-2/Beclin-1 axis [14]. These articles focus on the research progress of lncRNA from MSC-exo in myocardial injury and provide ideas for the repair of myocardial injury. In end-stage dilated cardiomyopathy, the levels of HAND2-AS1 is reduced and its tendency is involved in the survival rate of patients [15]. However, no researchers have paid attention to whether HAND2-AS1 plays an intermediary role in the influence of exos on H/R. Therefore, this study analyzed the protective impacts of exo from bone marrow mesenchymal stem cell (BMSC) in the H/R injury model of myocardial cells and its potential mechanism. The effect of HAND2-AS1 on cell viability, apoptosis, oxidative stress, and inflammation was deeply studied to prove whether HAND2-AS1 is involved in the beneficial effects of exo on H/R.

## Materials and methods

### Exosome isolation and collection

The culture supernatant of bone marrow mesenchymal stem cells (BMSC, CRL-12,424, ATCC, USA) was collected, and exos were extracted according to the instructions of the exo isolation kit (YEASEN, Shanghai, China). The specific steps were as follows. The culture supernatant was transferred to a centrifuge tube and centrifuged. The cell debris was removed and transferred to a new centrifuge tube. The reagent was added according to the ratio of cell supernatant: exo separation reagent = 4:1. After being mixed using a vortex, the reagent was incubated at 4℃ for two hours, and centrifuged at a low temperature of 10,000 g. After 60 min, the supernatant was discarded to keep the precipitation. The exo was resuspended with 100 µl PBS, and stored in the refrigerator at -80℃ for future use. The ethics committee of The Third Affiliated Hospital of Qiqihar Medical University provided the approval for this study.

Exo-specific protein CD63 and TSG101 were assessed to identify the exo. The exo sample and the lysis solution were added in a volume ratio of 1:1 and mixed evenly. The mixed culture was put on ice for lysis for 10 min. The supernatant was taken after being centrifuged at 4℃ for 5 min. The protein concentration was evaluated by BCA Protein Assay Kit (TIANGEN, Beijing, China) and Western Blot. A proper amount of protein samples was taken for SDS-PAGE. The diluted primary antibodies were added after being transferred to film and sealed. The diluted horseradish peroxidase (HRP) labeled secondary antibody was mixed and incubated for 2 h on a shaking table. The sample was developed with ECL and fixed in a dark room. Quantity One analysis of protein band gray value. The rabbit CD63 and anti-TSG101 antibodies were from Abcam (Cambridge, MA, USA).

### Establishment of H/R model

Myocardial cell H9c2 (ATCC, Rockville, MD, USA) in logarithmic growth phase were inoculated into a 24-well culture plate containing serum-free and sugar-free medium. The culture plate was cultured in an incubator containing 95% nitrogen and 5% carbon dioxide for 6 h and then replaced with a normal culture medium and cultured in an incubator containing 5% carbon dioxide [16]. H9c2 cells were treated using exo (5 µg of total protein) at 37˚C for 48 h to study the protective roles of exo.

H9c2 cells in logarithmic growth phase were collected and transfected with artificial sequences of HAND2-AS1 and miR-17-5p (10 pmol/mL, GenePharm, Shanghai, China) by Lipofectamine 3000 (Invitrogen, Carlsbad, CA, USA).

### The expression level was detected by real-time quantitative PCR(RT-qPCR)

Total RNA of H9c2 cells and exos was extracted by Trizol method (Invitrogen, Carlsbad, California, USA), and cDNA was synthesized by reverse transcription kits (Applied Biosystems, Foster City, CA, USA). The qRT-PCR reaction system was prepared using cDNA as a template. The relative expression of miR-17-5p and HAND2-AS1 was detected by a real-time PCR instrument (7500, ABI, Foster City, CA, USA). After calibration with U6 or β-actin, the concentration was calculated by the 2 ^−∆∆CT^ method. The primers were as follows: HAND2-AS1 forward 5’-CTCACCTCCCAGACCCT-3’ and reverse, 5’ ACAAGCCAACCCGTAAT-3’; β-actin forward 5’-GGCACCCAGCACAATGAA-3’ and reverse, 5’TAGAAGCATTTGCGGTGG-3’; miR-17-5p forward 5’-TCTAGATCCCGAGGACTG-3’ and reverse, 5’-ATCGTGACCTGAACC-3’; U6, forward 5’-CTCGCTTTGGCAGCACA-3’ and reverse, 5’-AACGCTTCACGAATTTGCGT-3’.

### MTT assay was utilized to evaluate cell viability

H9c2 cells in each group were transferred to a 96-well plate containing 10 µL of MTT reagent (Sigma, St. Louis, MO, USA). This plate was incubated for 4 h, added 150 µL of DMSO, and shaken for 10 min. The absorbance value (OD) of each well at 490 nm was detected by an enzyme-labeled instrument.

### Cell apoptosis was estimated by flow cytometry

The cells in each group were resuspended with 1 × binding buffer to adjust their concentration to 1 × 10^5^ cells/ml. 100 µL cell suspension was sequentially added with 5 µL Annexin V/FITC and 10 µL PI solution (Solarbio, Beijing, China) and incubated in the dark for 15 min. 1× binding buffe was added and the apoptotic result was estimated by flow cytometry.

### The contents of LDH, SOD, and MDA

After being treated, the cell culture solution was collected. The contents of LDH were detected according to the operating instructions of the kit (Jiangcheng, Nanjing, China). At the same time, H9c2 cells in each group were collected and lysed on ice for 30 min, centrifuged at 12,000 r/min at low temperature for 15 min. The supernatant was collected and the levels of SOD and MDA were detected according to the instructions of the kit (Beyotime, Shanghai, China).

### Luciferase reporter activity

WT-HAND2-AS1 carriers and MUT-HAND2-AS1 carriers were constructed by inserting HAND2-AS1 3’-UTR fragment or mutate HAND2-AS1 fragment into luciferase reporter gene vectors. The luciferase vectors of Mfn2 were constructed by the same method as that of HAND2-AS1. The vectors and miR-17-5p mimics were co-transfected into H9c2 cells, respectively. The transfected cells were collected for 48 h and the luciferase activity in each group was measured.

### Statistical analysis

Graphpadprism 7.0 software was used for statistical processing and drawing. One-way ANOVA was used for comparison among groups, and Tukey test was utilized for multiple comparisons between groups. Data are presented as mean values of independent experimental triplicates; error bars represent the standard deviation of the data. *P* < 0.05 is statistically significant.

## Results

### Effects of BMSC-derived exos on miR-17-5p expression, cell viability, and apoptosis rate of H/R cardiomyocytes

**Fig. 1:**
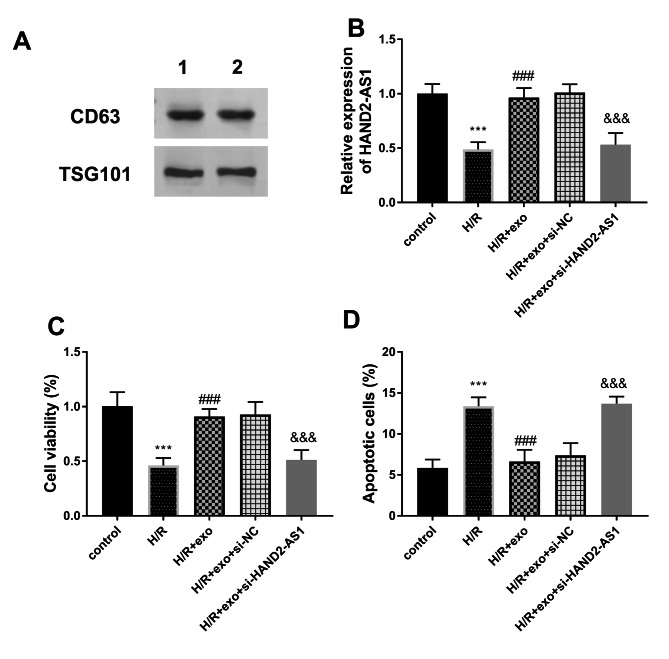
**(A)** The expression of CD63 and TSG101 protein was detected. **(B)** The expression of HAND2-AS1 was reduced in H/R group and its levels were regulated by exo and si-HAND2-AS1. **(C-D)** The viability and apoptosis were manipulated by exo and the concentration of HAND2-AS1. ***P < 0.001, relative to control group; ###*P* < 0.001, relative to H/R group; &&&*P* < 0.001, relative to H/R + exo group

The western blot results in Fig. 1A documented that the indicators CD63 and TSG101 were positive, suggesting the success of exo isolation. In H/R group, the amount of HAND2-AS1 in cardiac myocytes decreased, which provided that the the expression of HAND2-AS1 was influenced by H/R damage (*P* < 0.001, Fig. 1B). The level of HAND2-AS1 in the H/R + exo was partly increased compared with that of the H/R group (*P* < 0.001, Fig. 1B). It was assumed that the exo regulated the expression of HAND2-AS1 in H9c2 cells and it might participate in the H/R damage by modulating HAND2-AS1. The transfection of si-HAND2-AS1 was an inhibitor of HAND2-AS1 in cardiomyocytes, highlighting the success of transfection (*P* < 0.001, Fig. 1B).

In addition, viability and apoptosis were used to verify the cell activity. As depicted in Fig. 1C, the cell viability was destroyed by H/R treatment and partially restored by exo, which indicated the protective effect of exo on cell viability (*P* < 0.001). However, the inhibition of HAND2-AS1 cancelled the beneficial function of exo (*P* < 0.001, Fig. 1C). The apoptosis rate of the H/R group decreased obviously, while the exo inhibited the influence of H/R on H9c2 (*P* < 0.001, Fig. 1D). The elimination of HAND2-AS1 reversed the low apoptosis rate of H/R + exo group, which reflected that HAND2-AS1 could mediate the exo-associated impacts on the myocardial injury.

### Effects of BMSC-derived exos on the contents of LDH, MDA, and SOD in H/R cardiomyocytes

**Fig. 2:**
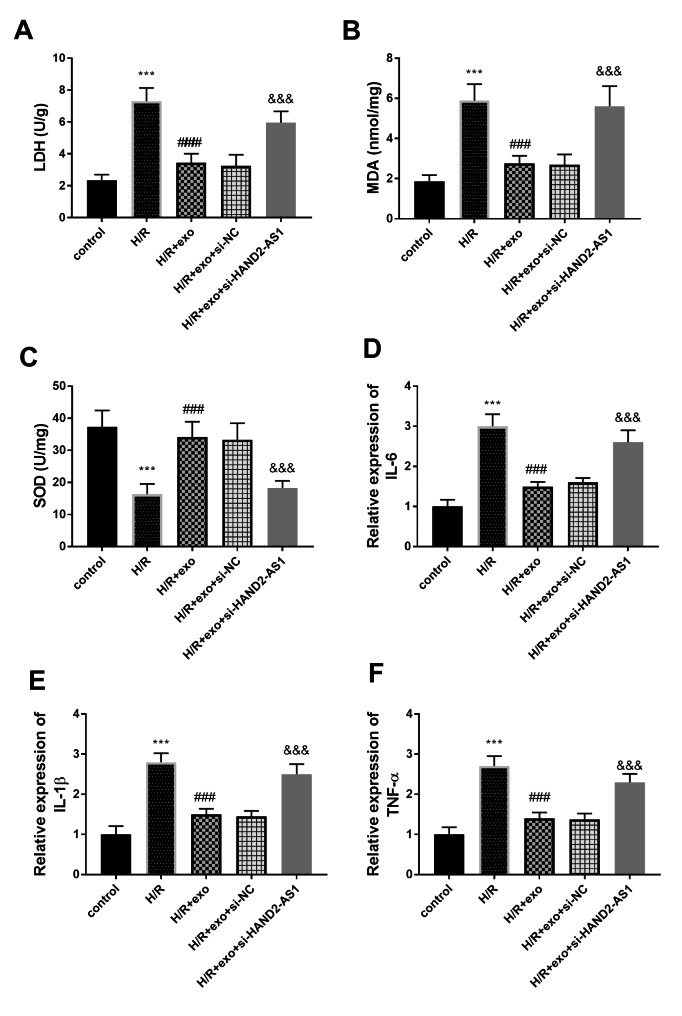
The impacts of exo and HAND2-AS1 on the content of **(A)** LDH, **(B)** MDA, **(C)** SOD, **(D)** IL-6, **(E)** IL-1β, and **(F)** TNF-α. ***P < 0.001, relative to control group; ###*P* < 0.001, relative to H/R group; &&&*P* < 0.001, relative to H/R + exo group

In H/R group, the contents of LDH and MDA in myocardial cells increased, but the secretion of SOD decreased (*P* < 0.001, Fig. 2A-C). The exo treatment protected H9c2 against H/R damage on oxidative stress, which has been confirmed by the evidence that exo reduced the LDH and MDA content and increased the SOD levels (*P* < 0.001, Fig. 2A-C). The leakage of LDH, MDA, and SOD changed after the transfection of HAND2-AS1, indicating that silent HAND2-AS1 prevented the effect of exo (*P* < 0.001, Fig. 2A-C).

### Effects of BMSC-derived exos on inflammation in H/R cardiomyocytes

The inflammatory balance of cardiomyocytes was reflected by the production of IL-6, IL-1β, and TNF-α. As shown in Fig. 2D-F, in the H/R group, the content of inflammatory cytokines was enhanced, indicating that H/R damaged the inflammatory balance and led to the inflammatory disorder (*P* < 0.001). The exo decreased the amount of inflammatory markers, while si-HAND2-AS1 limited the influence of exo (*P* < 0.001, Fig. 2D-F).

### HAND2-AS1 targeted regulation of mir-17-5p expression

**Fig. 3:**
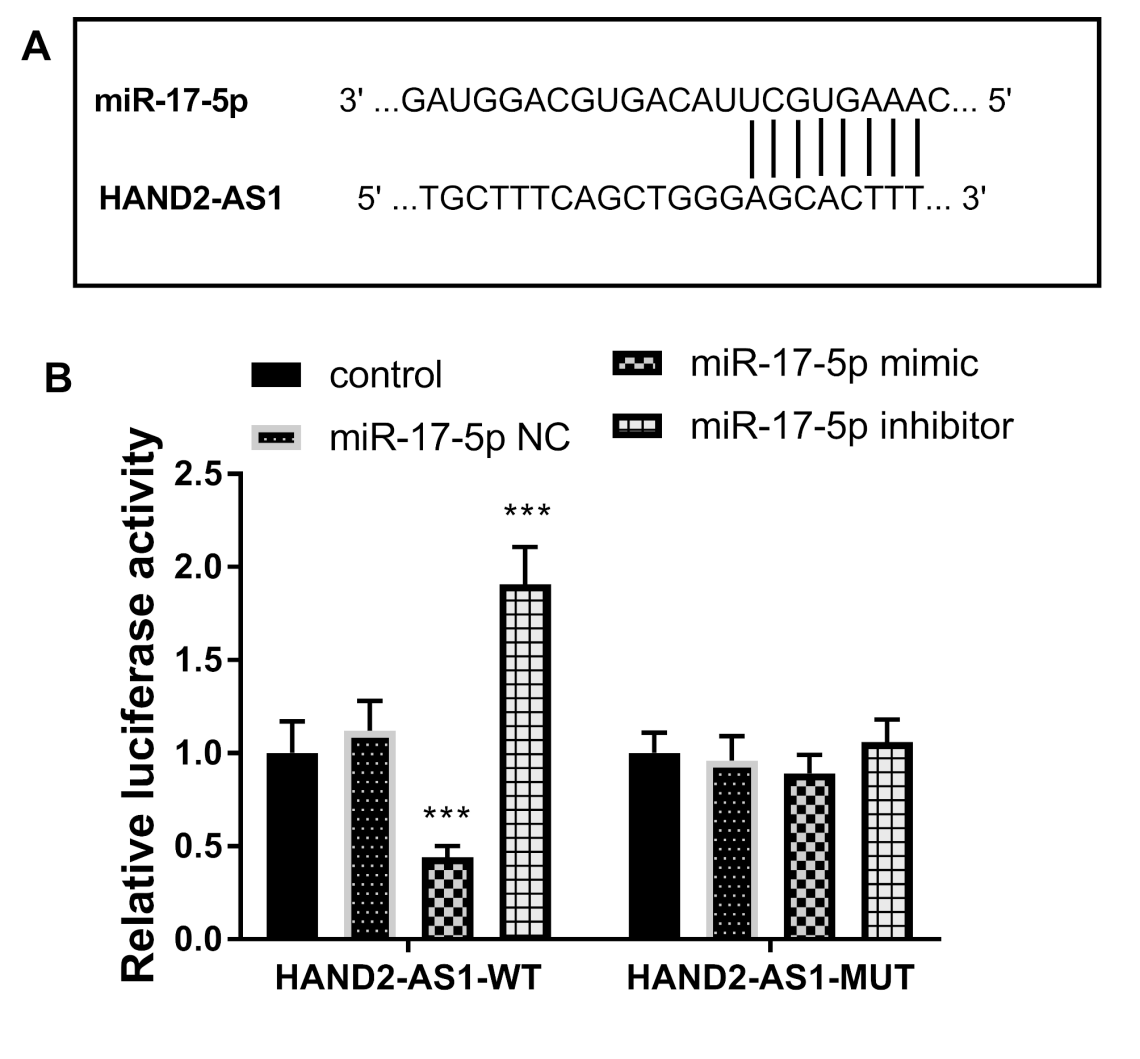
**(A)** The putative sites between miR-17-5p and HAND2-AS1. **(B)** The targeted correlation was verified by luciferase activities

There are some continuous binding bases between miR-17-5p and 3’UTR of HAND2-AS1 sequence, as shown in Fig. 3A. When the expression of miR-17-5p was up-regulated or down-regulated, the luciferase activity of cardiomyocytes transfected with WT-HAND2-AS1 decreased or increased significantly (*P* < 0.001, Fig. 3B), while cardiomyocytes transfected with MUT-HAND2-AS1 did not change obviously (*P* > 0.05, Fig. 3B).

### Overexpression of mir-17-5p reverses the effect of HAND2-AS1 on the injured cardiomyocytes induced by H/R

**Fig. 4:**
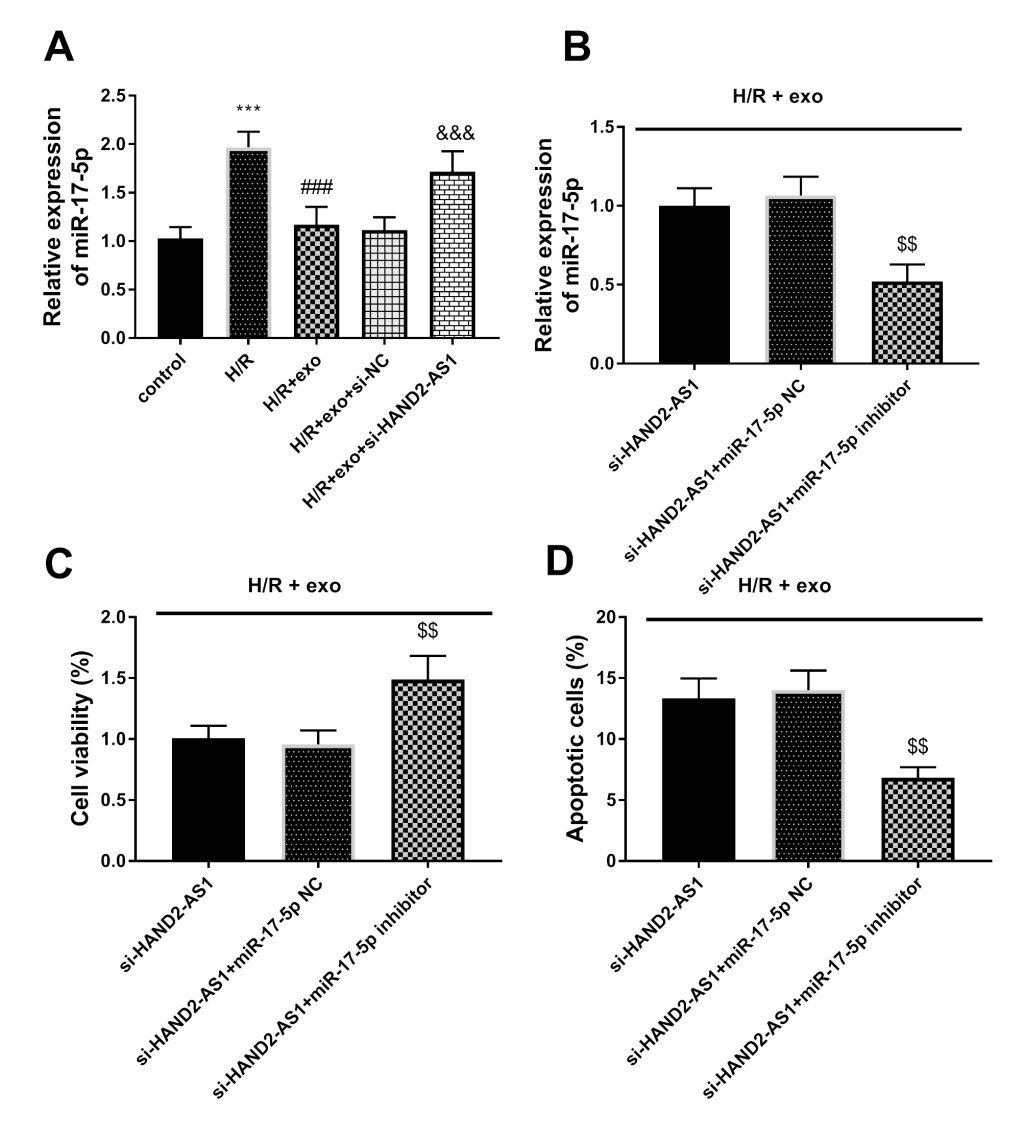
**(A)** The relative levels of miR-17-5p were elevated in the H/R group, reduced in the H/R + exo group, and partially restored in the H/R + exo + si-HAND2-AS1 group. **(B)** The transfection of miR-17-5p inhibitors restricted the content of miR-17-5p. **(C-D)** The influence of MiR-17-5p on survival rate and apoptotic rate

In light of the interconnection of miR-17-5p and HAND2-AS1, the mediated function of miR-17-5p was studied. The content of miR-17-5p was increased in the H/R group and exo inhibited the elevated expression of miR-17-5p (*P* < 0.001, Fig. 4A). When the expression of HAND2-AS1 was down-regulated, the expression of miR-17-5p was increased (*P* < 0.001, Fig. 4A).

**Fig. 5:**
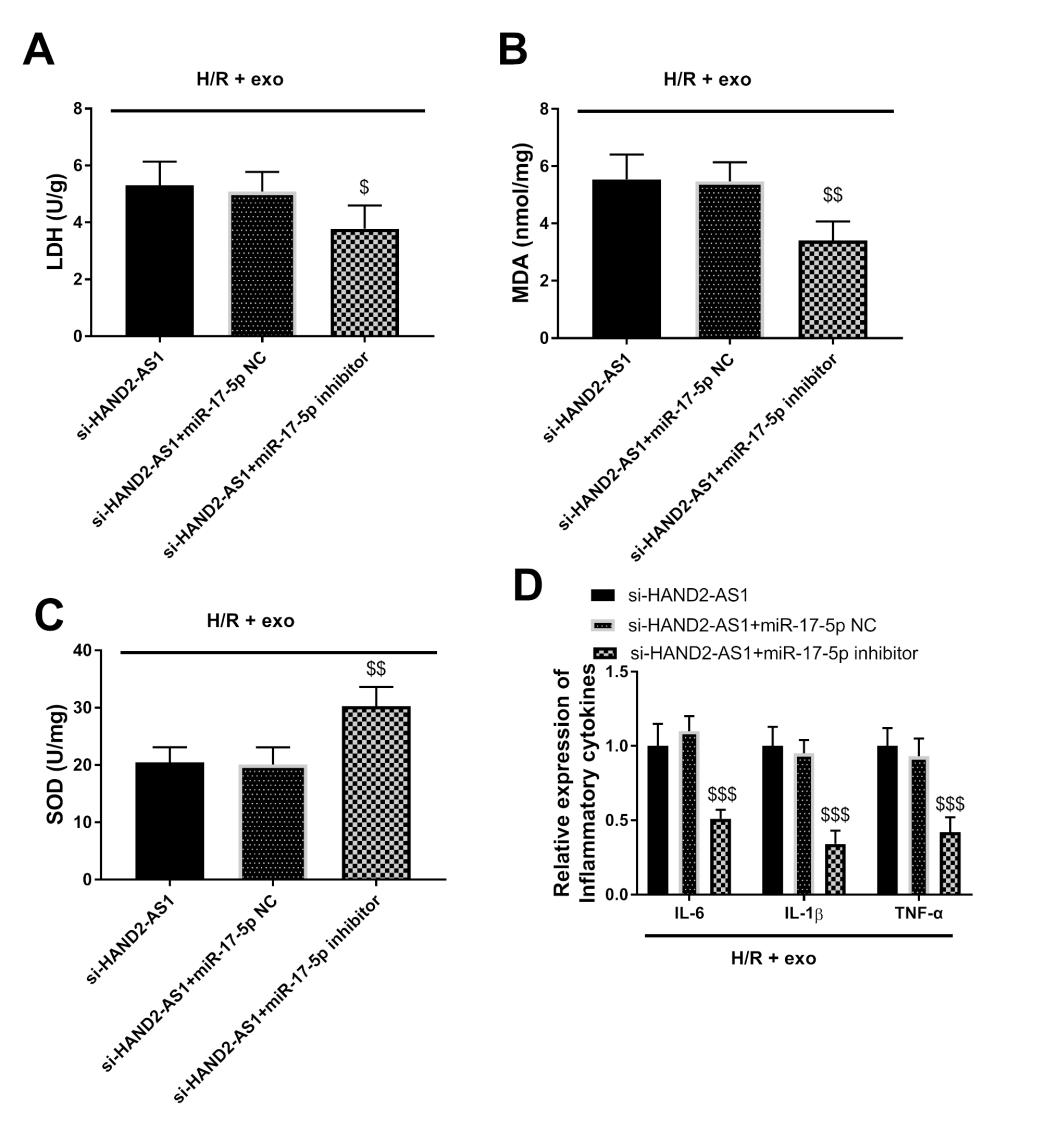
MiR-17-5p accommodated the **(A-C)** oxidative situation and **(D)** inflammation of cardiomyocytes

In all cells treated with H/R + exo cells, the expression of miR-17-5p in the si-HAND2-AS1 + miR-17-5p inhibitor group was declined relative to the si-HAND2-AS1 group (*P* < 0.01, Fig. 4B). The declined miR-17-5p elevated the cell viability and suppressed the number of apoptotic cells (*P* < 0.01, Fig. 4C-D), corroborating that miR-17-5p participated in the regulation of H/R damage. In H/R and exo-treated cells, the oxidation was regulated by the deficiency of miR-17-5p, which was reflected in the fact that knockdown of miR-17-5p reduced LDH and MDA, and increased SOD levels (*P* < 0.05, Fig. 5A-C). The expression of inflammatory markers was attenuated by the absence of miR-17-5p (*P* < 0.001, Fig. 5D). Taken together, the HAND2/AS1 pathway functioned as an avenue in the protection of exo on myocardial cells induced by H/R.

### MiR-17-5p targeted regulation of Mfn2 expression

**Fig. 6:**
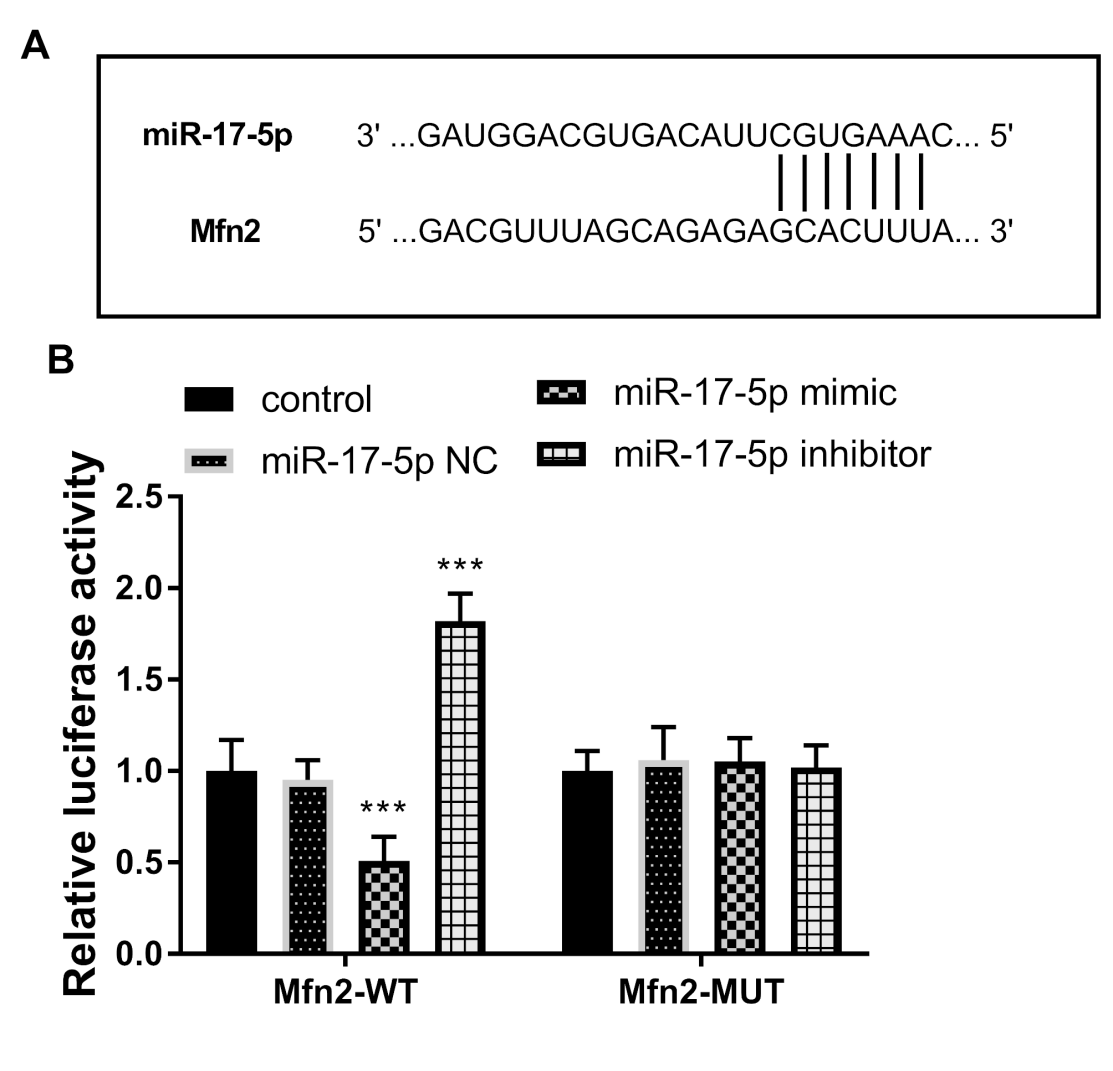
**(A)** The putative sites between miR-17-5p and Mfn2. **(B)** The finding of luciferase activities

Bioinformatics predicts that Mfn2 might be induced by miR-17-5p. The possible binding sites are shown in Fig. 6A. Subsequently, we further verified their relationship by luciferase reporter assay. When mimics or inhibitors of miR-17-5p were co-transfected with Mfn2-WT, the signal of miR-17-5p mimic group was weaker than that of control group, miR-17-5p inhibitor group was enhanced (*P* < 0.001, Fig. 6B).

## Discussion

AMI is a cardiovascular disease with a high mortality rate [17]. Clinically, thrombolysis and coronary artery bypass surgery are mainly used to restore blood supply to myocardium [18, 19]. However, once the blood supply of myocardium is restored, it will cause oxidative stress in myocardial cells, leading to cell injury and apoptosis [20]. Therefore, it is of great significance to study how to alleviate myocardial ischemia-reperfusion injury.

Exos are vesicles secreted by cells with phospholipid bilayer structure. Exo can transport proteins and other cytokines to recipient cells and mediate cell-to-cell communication under physiological and pathological conditions [21]. Exos are involved in intercellular communication and intercellular macromolecular transport [22]. MSCs play a therapeutic role by suppressing inflammation, decreasing apoptosis, and promoting angiogenesis through certain differentiation [23]. Studies believe that MSCs inhibit inflammation and apoptosis and promote angiogenesis. This function is realized mainly through its secretory action [24]. Thus, this study utilized cardiomyocytes as models to research the impacts of BMSC-derived exo on H/R. The myocardial H/R model was constructed, treated by exo, and transfected with artificial sequences. The function of HAND2-AS1 delivered by exos from BMSC on myocardial models was explored. Myocardial injury models significantly reduced the survival rate and increased the apoptosis rate of H9c2 cells, while the exo treatment restored cell viability and inhibited cell death rate, which highlighted the protective effect of exo on H/R damaged H9c2. Oxidative stress and inflammation are important factors in myocardial injury and play vital roles in AMI [25, 26]. According to the results of this study, exo in myocardial cells damaged by H/R could reduce oxidative stress and inflammatory reaction. It was concluded that exo might protect cardiomyocytes from H/R injury by improving cell survival rate and limiting oxidative stress and inflammation.

The role of exo-derived lncRNAs in cardiovascular diseases attracts researchers’ interest. Many lncRNAs, including lncRNA ZFAS1 and MALAT1, are involved in the potential mechanism of heart trauma [27, 28]. Extracellular LINC00174 produced by endothelial cells inhibits p53-mediated autophagy and apoptosis, so as to alleviate myocardial injury caused by I/R [29]. By binding miR-556-5 p/XIAP, lncRNA A2M-AS1 derived from MSC-derived exos weakened the apoptosis and oxidation injured by H/R [30]. Current research results provided that HAND2-AS1 was a regulator of myocardial injury. The deficiency of HAND2-AS1 reduced cell survival rate, expedited cell apoptosis, exaggerated oxidative stress, and stimulated inflammatory indicators. HAND2-AS1 partially eliminated the protective effects of exo. HAND2-AS1 delivered by exo of MSCs is reduced in rheumatoid arthritis, and its regeneration inhibited the expression of inflammatory indicators, which certified our finding about the effect of HAND2-AS1 on inflammation [31]. Therefore, exo played a beneficial role through HAND2-AS1.

Many reports indicate that miRNAs are differentially expressed in cardiovascular diseases and play important roles in many aspects. The expression of miR-17-5p was elevated in myocardial infarction, and inhibition of miR-17-5p restricted the apoptosis of cardiomyocytes [32]. The miR-17-5p delivered by exos regulated the progression and inflammation of the abdominal aortic aneurysm [33]. A study investigated the human lung treated with isolated lung perfusion and found that miR-17-5p was up-regulated after perfusion [34]. In this study, the targeted relationship between miR-17-5p and HAND2-AS1 was confirmed. The influence of inhibited HAND2-AS1 on cell viability, cell apoptosis, oxidative stress, and inflammation was attenuated by miR-17-5p inhibitors, reflecting the mediated effects of miR-17-5p on HAND2-AS1. Therefore, exo regulated the HAND2-AS1/miR-17-5p pathway, thus protecting the activity of cardiomyocytes. Yang et al. prove the interaction of miR-17-5p and HAND2-AS1 in bladder cancer [35]. MicroRNA plays an important role in myocardial ischemia-reperfusion injury by regulating some genes. This observation lends support to the fact that Mfn2 was a target of miR-17-5p. Mfn2 is a mitochondrial fusion protein, which is widely researched in myocardial dysfunctions. In pathological cardiac hypertrophy, miR-17-5p can accelerate the degeneration of cellular hypertrophy by mediating Mfn2 [36]. Short of Mfn2 increased mitochondrial damage and oxidative stress [37]. Zhao et al. report that the protein expression of Mfn2 is lowly expressed in H9c2 cells after H/R treatment [38], which shows its expression opposite to miR-17-5p. Therefore, the HAND2-AS1/miR-17-5p/Mfn2 axis was related to the protective effect of exo on myocardial function. However, this study did not consider the different functions of MSC in various species and did not carry out design using cardiomyocytes or BMSCs from different species.

## Conclusion

To sum up, the exo from BMSC could alleviate the damage of H/R to H9c2 cells, and its protective effect on exo was related to the activation of the HAND2-AS1/miR-17-5p/Mfn2 pathway. HAND2-AS1 might protect cardiomyocytes from H/R-induced viability, apoptosis, oxidative stress, and inflammation by targeting the expression of miR-17-5p.

## Electronic supplementary material

Below is the link to the electronic supplementary material.

Supplementary Material 1: Original western blot images displayed in Fig. 1A.

## Data Availability

All data generated or analyzed during this study are included in this article. Further enquiries can be directed to the corresponding author.
