# Supplementary material for: Protective effect of bone marrow mesenchymal stem cell-derived exosomes on cardiomyoblast hypoxia-reperfusion injury through the HAND2-AS1/miR-17-5p/Mfn2 axis
Source: BMC Cardiovasc Disord. 2023 Mar 7;23:114. doi: 10.1186/s12872-023-03148-4 (PMC9993697; doi:10.1186/s12872-023-03148-4)
Supplement: Supplementary file 1 — Supplementary Material 1: Original western blot images displayed in Fig. 1A. [file 12872_2023_3148_MOESM1_ESM.pdf]

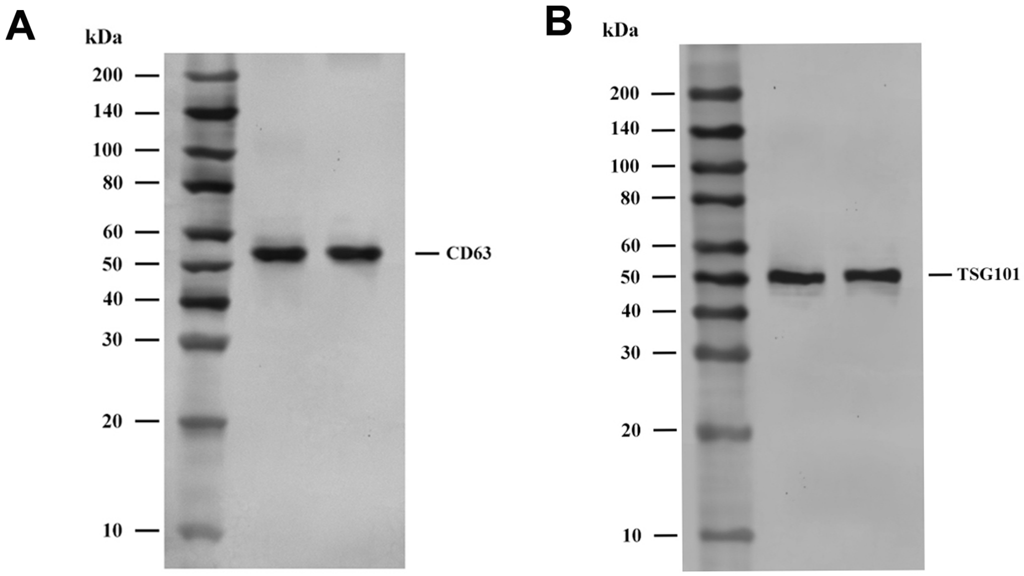

Supplementary Figure 1. The original bands of western blot in Figure 1A. Western blot images of (A) CCD63 and (B) TSG101 in Figure 1A.
